# Prevalence of Hyponatremia in Femur Neck Fractures: A One-Year Survey in an Urban Emergency Department

**DOI:** 10.1155/2014/397059

**Published:** 2014-09-15

**Authors:** Gianfranco Cervellin, Michele Mitaritonno, Mario Pedrazzoni, Alessandra Picanza, Giuseppe Lippi

**Affiliations:** ^1^Emergency Department, Academic Hospital of Parma, Via Gramsci 14, 43126 Parma, Italy; ^2^Department of Internal Medicine, University of Parma, 43126 Parma, Italy; ^3^Laboratory of Clinical Chemistry and Hematology, Academic Hospital of Parma, 43126 Parma, Italy

## Abstract

This study was aimed at investigating the prevalence of hyponatremia in patients with intracapsular femoral neck fracture. All records containing clinical and laboratory information of patients admitted with femoral neck fractures to the Academic Hospital of Parma (Italy) during the year 2013 were retrieved from the hospital database. The control population consisted of subjects admitted to the outpatient phlebotomy center during the same period. The final population consisted of 543 patients with femoral neck fractures and 700 outpatients. The category of elderly subjects (i.e., ≥65 years) included 491 patients and 380 controls. In both the entire population and elderly subjects, serum sodium was lower in patients than in controls (138 versus 139 mmol/L, *P* < 0.001). The prevalence of hyponatremia was also higher in cases than in controls, both in the entire population (19.5 versus 10.4%, *P* < 0.001) and in elderly subjects (20.8 versus 11.8%, *P* < 0.001). The odds ratio of hyponatremia for femoral neck fracture was 2.08 in the entire study population and 1.95 in those aged 65 years and older. In conclusion, we found that hyponatremia is significantly associated with femoral neck fracture. Serum sodium should hence be regularly assessed and hyponatremia eventually corrected.

## 1. Introduction

The falls should now be regarded as a major public health issue [1]. Approximately 424,000 fatal falls occur annually worldwide, thus representing the second leading cause of death due to accidental injury after road traffic collisions [2]. Single and repeated falls are a special health concern in the elderly. According to recent European surveys, at least 20% of people aged 65 years or older suffer at least one fall per year [3], and up to 20% of falls result in a significant injury in the elderly [4].

The falls are at least in part preventable, especially in the elderly [1]. The origin of the fall is typically multifactorial and includes environment-related aspects, along with person- and behaviour-related factors. Among these, gait instability has been identified as a relatively consistent risk factor [5]. Several drugs, most notably psychotropic drugs, antihypertensive drugs, anticonvulsants, or multiple medications, have also been significantly associated with the risk of fall [6, 7]. Femur neck fractures represent one of the most serious consequences of falls in the elderly, carrying a significant risk of morbidity [8] and mortality, with the latter approximating a rate 13.5% at 6 months [9].

Several lines of evidence also attest that hyponatremia is a frequent disorder in the elderly, affecting up to 10% of hospitalized patients [10]. It is noteworthy that up to one-quarter of hyponatremic patients seek initial medical treatment in the Emergency Department (ED) [11]. Approximately 50% of these cases are due to the syndrome of inappropriate antidiuretic hormone secretion (SIADH), and the remaining rate is iatrogenic in nature (most notably diuretics, psychotropic drugs, and anticonvulsants) or is associated with chronic conditions such as hypothyroidism, congestive heart failure, liver cirrhosis, and renal failure [12].

Hyponatremia, generally defined as a serum sodium concentration <136 mmol/L [13], has been recently associated with gait disturbances, falls [14], and fractures in the elderly [15–17]. In one single study including elderly patients with femur neck fracture, the prevalence of hyponatremia was found to be more than 3-fold higher in these patients compared to 44 ambulatory patients admitted with elective hip or knee prosthesis (i.e., 16.9% versus 4.6%) [18]. As such, the aim of this study was to investigate the prevalence of hyponatremia in all consecutive femoral neck fracture patients admitted to our hospital during the year 2013.

## 2. Materials and Methods

All records containing clinical and laboratory information of patients admitted with femoral fractures to the Academic Hospital of Parma (Italy) during the year 2013 were retrieved from the local hospital database. All patients have been admitted within 12 hours from trauma. All the patients with extracapsular, diaphyseal, or distal femur fractures were then excluded, so that only femur neck fractures were considered. All the blood tests, sodium included, have been obtained at presentation in ED, before the administration of intravenous rehydration. The control population consisted of 700 outpatients consecutively refereed for routine testing to the outpatient phlebotomy center of the same hospital and during the same period. Sodium was routinely measured in both populations in serum, by an indirect ion-selective electrode (ISE) method and using the same analytical instrumentation (i.e., Beckman Coulter AU5800; Beckman Coulter Inc., Brea, CA, USA). The total imprecision of this method was found to be lower than 0.8% [19]. The quality of serum sodium measurement was validated by regular internal quality control procedures and participation in External Quality Assessment Scheme throughout the study period.

For the purposes of this investigation, hyponatremia has been defined as a serum sodium concentration <136 mmol/L, whereas severe hyponatremia has been defined as a serum sodium concentration <125 mmol/L, according to widespread consensus [13]. The serum sodium values obtained at admission were selected for patients with femur neck fractures, thus ahead of establishing in-hospital therapy or undergoing surgery. Results were finally expressed as median and interquartile range (IQR). Differences between groups were assessed with Wilcoxon-Mann-Whitney test (for continuous variables) and *χ* ^2^ test with Yates' correction (for categorical variables), using Analyse-it (Analyse-it Software Ltd., Leeds, UK). The odds ratios (ORs) were calculated using MedCalc Version 12.3.0 (MedCalc Software, Mariakerke, Belgium). The predictive value of hyponatremia was also investigated by means of receiver operating characteristic (ROC) curve. The study was performed in accordance with the Declaration of Helsinki, under the terms of relevant local legislation.

## 3. Results

After exclusion of noneligible cases (i.e., extracapsular, diaphyseal, or distal femur fractures; *n* = 178), the final study population consisted of 543 patients with femoral neck fractures. Patients and controls were also partitioned into two groups according to their age, that is, <65 years or ≥65 years. The latter category (i.e., ≥65 years) included 491 patients with femoral neck fractures and 380 controls.

**Figure 1:**
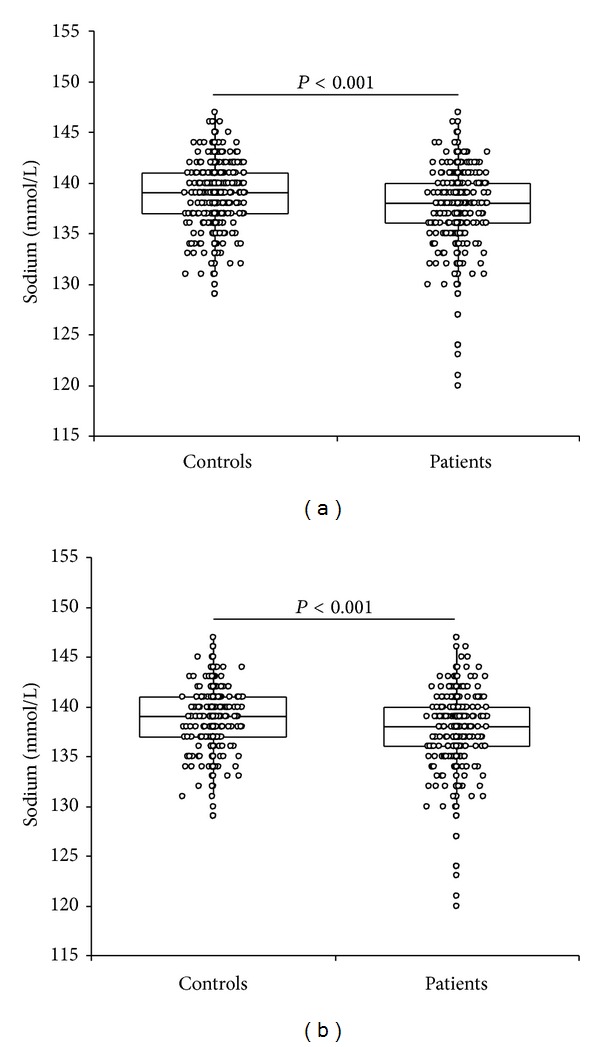
Serum sodium concentration in patients with femur neck fracture and in a control population of unselected outpatients. (a) Whole population and (b) subjects aged 65 years or older.

The results of the study, along with the basic demographic data, are shown in Table 1. In the entire population a significant difference was found in the concentration of serum sodium between patients and controls (138 versus 139 mmol/L, *P* < 0.001) (Figure 1(a)). The calculated mean percentage difference between groups (i.e., 0.87%) was marginally higher than the interindividual biologic variation of serum sodium (i.e., 0.7%) [20]. The observed difference (i.e., 1 mmol/L) was also higher than the minimal clinically important difference for serum sodium calculated in both populations (i.e., 0.15 mmol/L in cases and 0.11 mmol/L in controls) according to Copay et al. [21]. Patients with femur neck fractures also displayed a higher prevalence of both mild and severe hyponatremia compared to controls (19.5% versus 10.4%, *P* < 0.001 for mild hyponatremia; 1.1% versus 0%, *P* = 0.009 for severe hyponatremia). Similar results were found after excluding subjects aged less than 65 years. In the elderly, the serum sodium concentration was in fact also lower in cases than in controls (i.e., 138 versus 139 mmol/L, *P* < 0.001) (Figure 1(b)), and patients with femur neck fractures displayed a higher prevalence of both hyponatremia (20.8% versus 11.8%, *P* < 0.001) and severe hyponatremia (1.2% versus 0%, *P* = 0.04). The rate of hypernatremia (i.e., serum sodium >145 mmol/L) was identical in cases and controls (i.e., 0.7% versus 0.7%; *P* = 0.38).

In patients aged 65 years or older the difference of serum sodium remained statistically significant between cases and controls in both males (138 mmol/L and IQR 136–140 mmol/L versus 139 mmol/L and IQR 137–140 mmol/L; *P* = 0.007) and females (138 mmol/L and IQR 136–140 mmol/L versus 140 mmol/L and IQR 138–141 mmol/L; *P* < 0.001).

The odds ratio (OR) of hyponatremia for femur neck fracture was 2.08 (95% CI, 1.51 to 2.88; *P* < 0.001) in the entire study population and 1.95 (95% CI, 1.34 to 2.85; *P* < 0.001) in those aged 65 years or older.

In ROC curve analysis, hyponatremia exhibited a highly significant area under the curve (AUC) for predicting femur fracture (0.61; 95% CI, 0.57 to 0.64; *P* < 0.001). More specifically, a value lower than 129 mmol/L displayed 1.00 positive predictive value and 0.57 positive predictive value for predicting femur fracture.

In multivariate analysis, in which femur fracture was entered as dependent variable whereas serum sodium, age, and sex were entered as independent variables, a lower value of serum sodium remained as a highly significant predictor of femur fracture (beta coefficient −0.021; *P* < 0.001).

## 4. Discussion

The World Health Organization Collaborating Centre for Metabolic Bone Diseases has recently developed a country-specific fracture risk index of clinical risk factors (FRAX), which estimates the 10-year probabilities of hip and other major osteoporotic fractures [22, 23]. The FRAX score comprises, as risk factors, femoral neck bone mineral density, prior fractures, parental hip fracture history, age, gender, body mass index, ethnicity, smoking, alcohol use, corticosteroids use, rheumatoid arthritis, and secondary osteoporosis [22, 23]. Other significant clinical risk factors (such as history of falls and some metabolic derangements) have not been included in the FRAX algorithms so far, due to the lack of validation in prospective cohorts. Therefore, both scientists and clinicians are in continuous search for additional risk factors, which may be capable of improving the prediction of falls in both the general population and the elderly [24, 25].

Some previous studies showed that the prevalence of hyponatremia may be significantly higher in elderly patients admitted with a fracture than those admitted with different problems [15–17] and, more specifically, only one study reported similar findings in patients with femur neck fractures [18].

Our results, obtained in the largest cohort of patients with intracapsular femur neck fracture available so far, are in support of these preliminary findings. Interestingly, we also found that the association between femur neck fracture and hyponatremia is independent of the gender but is significant both in the general population and in the elderly, displaying a remarkable OR of approximately 2 (2.08 in the general population and 1.95 in the elderly).

Owing to the recent publication of data showing that hyponatremia may directly influence bone metabolism and can hence represent an important predisposing factor for osteoporosis and skeletal frailty [26], this electrolyte disorder should hence be regarded not only as a direct contributor of osteoporosis, but also as an important factor for impairing gait, finally leading to falls [27].

It is still uncertain whether hyponatremia should be considered a simple epiphenomenon of increased risk for femoral neck fracture, or rather a predisposing factor for falls and fractures in the elderly. Moreover, data are still lacking on how this clinical information should be used. Regardless of the causal or casual nature of this association, the concentration of serum sodium should be regarded as part of a complex and multifactorial impairment that increases the risk of gait disturbances and osteoporosis in the elderly. Accordingly, serum sodium levels should be regularly assessed and eventually corrected when the value falls below the lower limit of the reference range.

Unfortunately, no studies have evaluated bone density or incidence of falls and fractures before and after correction of hyponatremia, and neither of the prospective investigations aimed to demonstrate potential improvements of clinical outcomes after correction of hyponatremia are available so far. Since the serum sodium is a simple, inexpensive, and rapid biochemical test, it seems reasonable to suggest that clinicians should investigate the presence of hyponatremia in all elderly patients undergoing therapies with drugs potentially involved in the pathogenesis of hyponatremia, in particular diuretics, psychotropic drugs, and anticonvulsants. Those patients with mild chronic hyponatremia should also be probably examined for bone mineral density (BMD) and for falls risk using the available predictive models.

With all that being said, the treatment of mild hyponatremia remains an open question, especially in the elderly. Several lines of evidence suggest that rapid correction of chronic hyponatremia may be associated with severe complications, especially serious neurologic injury in patients undergoing fluid restriction [28]. A safer strategy may be represented by the administration of a new class of drugs, the vasopressin antagonists or vaptans, which are capable of correcting hyponatremia in different clinical conditions, although data on major clinical outcomes are still conflicting and somewhat lacking [29].

A potential limitation of this study is represented by the lack of data on the aetiology of hyponatremia in both cases and controls. It is noteworthy, however, that a recent study reported that the vast majority of elderly patients, including those with fragility fractures, had hyponatremia with multifactorial aetiology, and no significant differences were found in specific causes of hyponatremia between those developing fragility fractures and those who did not [30]. As such, regardless of the specific cause, the significance of the association found between hyponatremia and intracapsular femur neck fracture remains substantial in our investigation.

The cross-sectional design is another potential limitation of this study, since both exposure and outcome were simultaneously assessed and evidence of a temporal relationship could not be established. Further longitudinal studies are hence necessary to definitely clarify the predictive role of low serum sodium in the epidemiology of femur fracture.

## Figures and Tables

**Table 1:** Serum sodium concentration in patients with femoral neck fracture and in a control population of unselected outpatients.

Total population	Controls	Patients	*P*
*n*	700	543	
Age (years)	66 (52–77)	84 (77–89)	<0.001
M/F	407/293	387/156	<0.001
Sodium (mmol/L)	139 (137–141)	138 (136–140)	<0.001
(i) Hyponatremia	73/700 (10.4%)	106/543 (19.5%)	<0.001
(ii) Severe hyponatremia	0/700 (0%)	6/543 (1.1%)	0.009

>65 years	Controls	Patients	*P*

*n*	380	491	
Age (years)	76 (71–83)	85 (80–90)	<0.001
M/F	223/157	360/131	<0.001
Sodium (mmol/L)	139 (137–141)	138 (136–140)	<0.001
(i) Hyponatremia	45/380 (11.8%)	102/491 (20.8%)	<0.001
(ii) Severe hyponatremia	0/380 (0%)	6/491 (1.2%)	0.040
